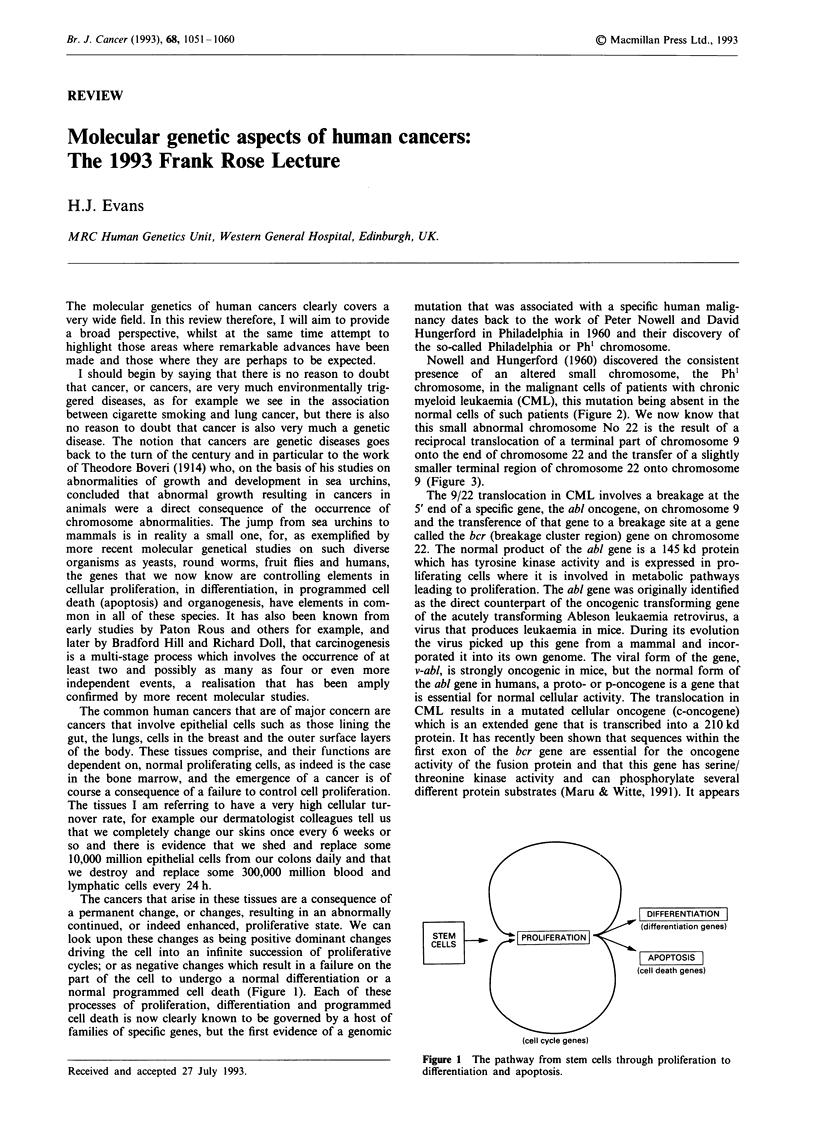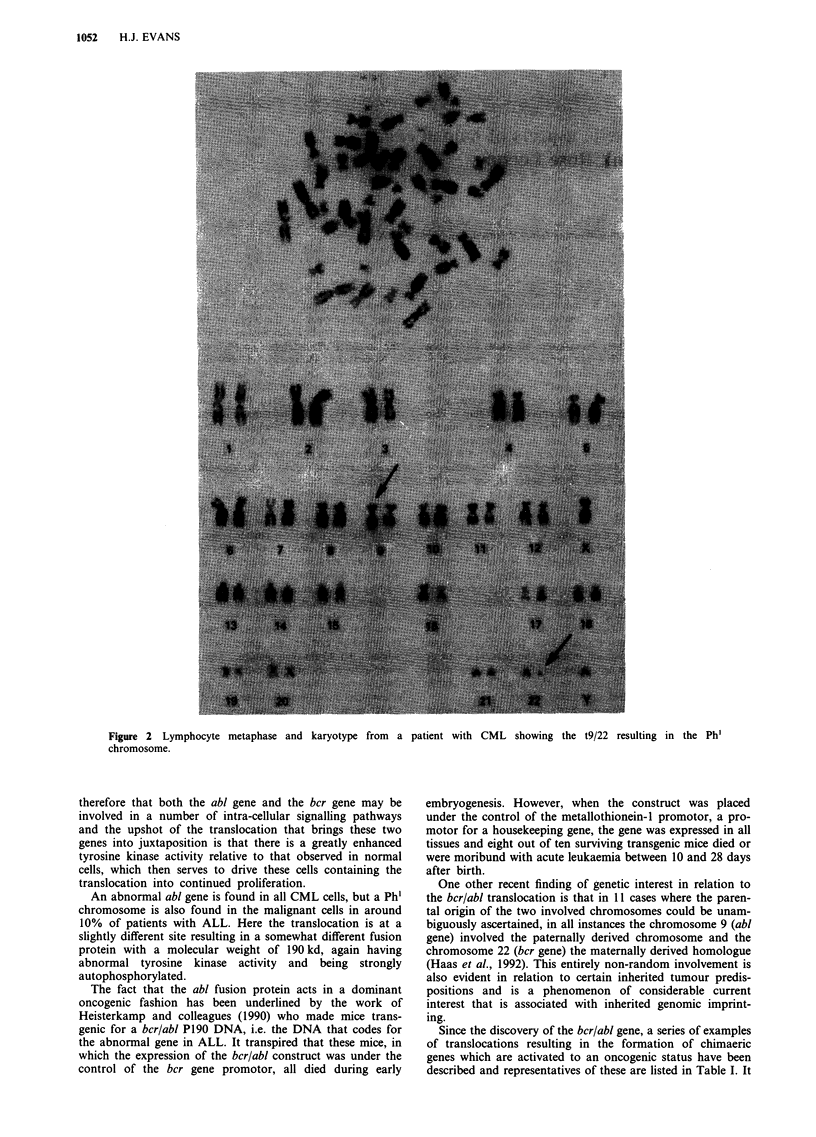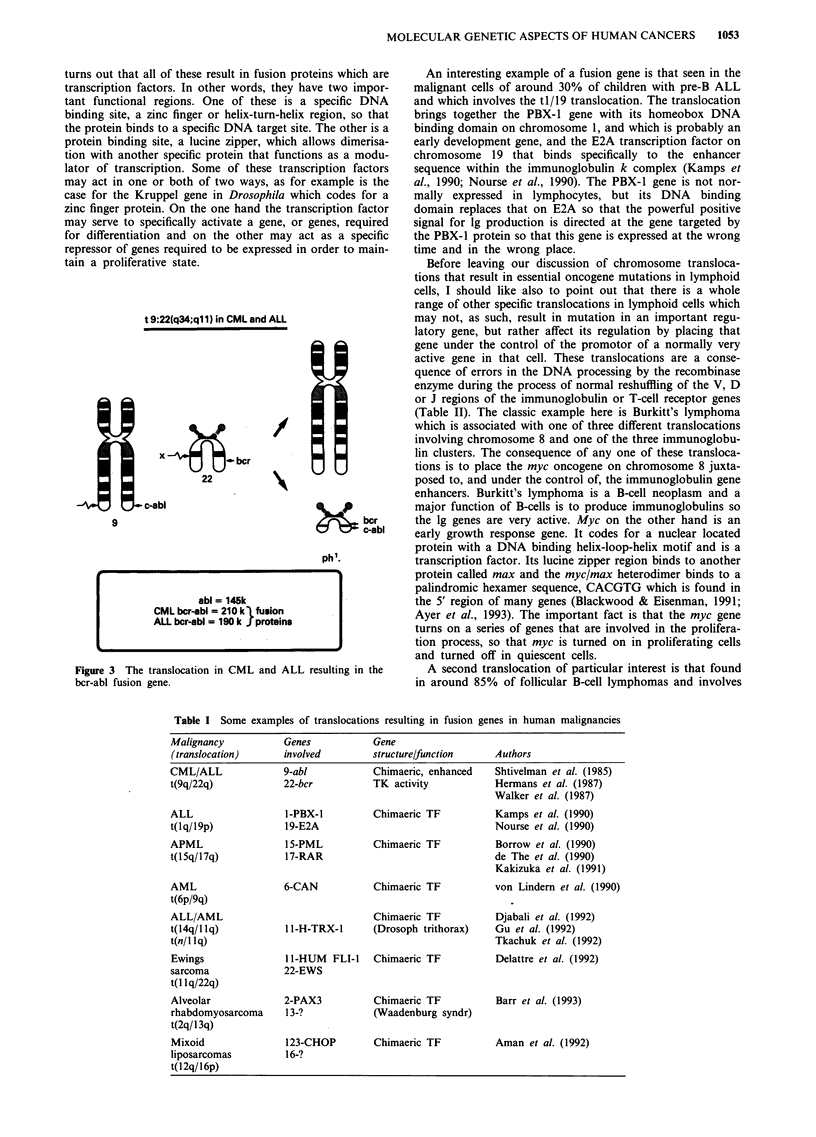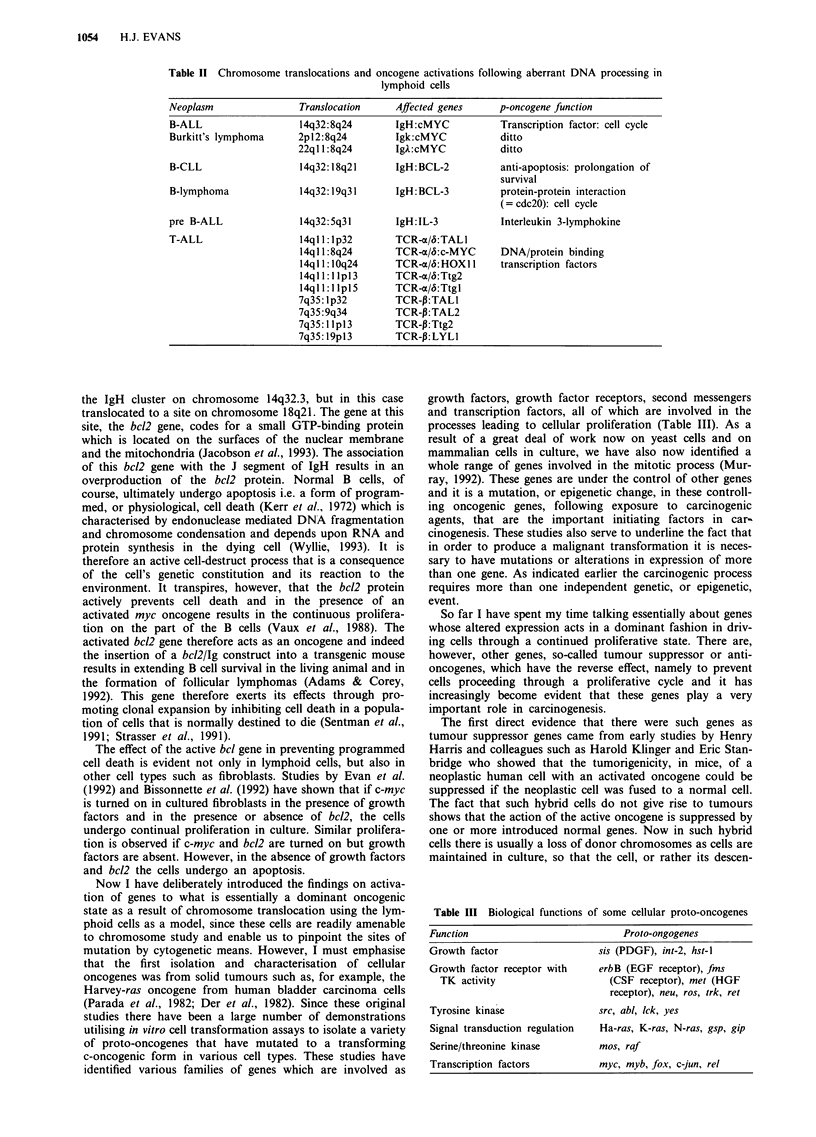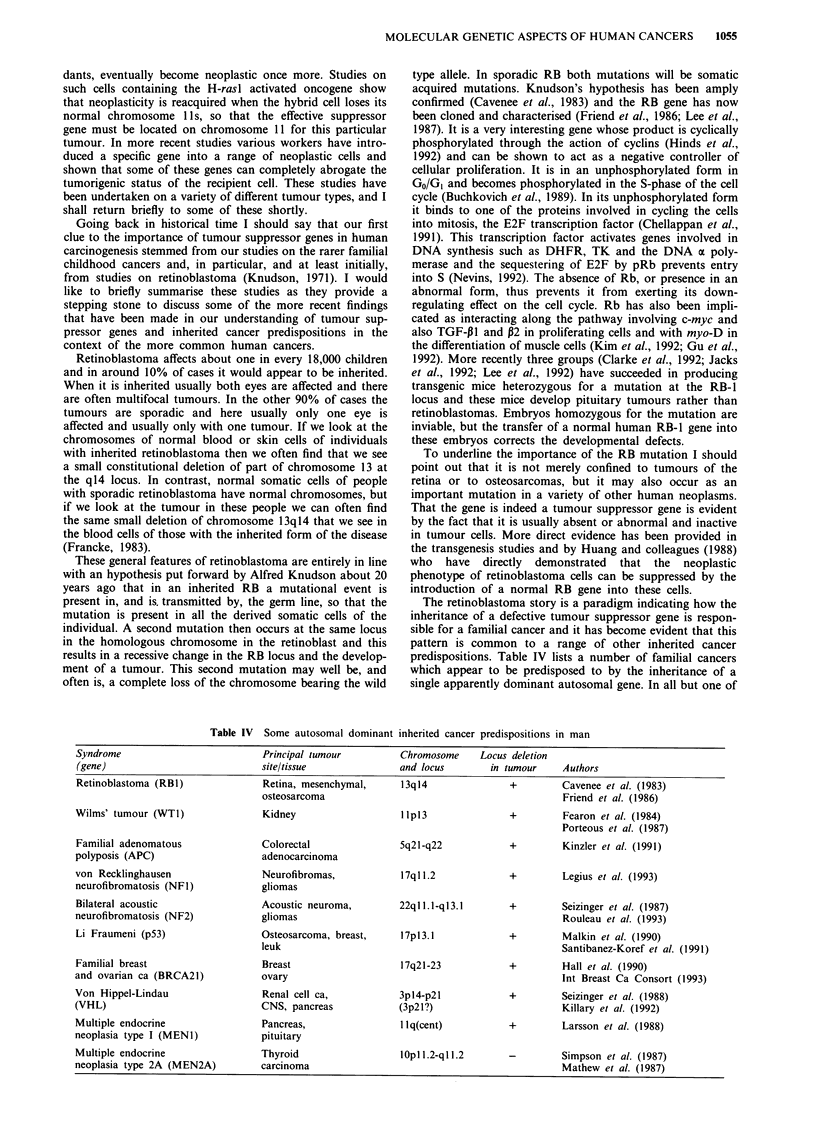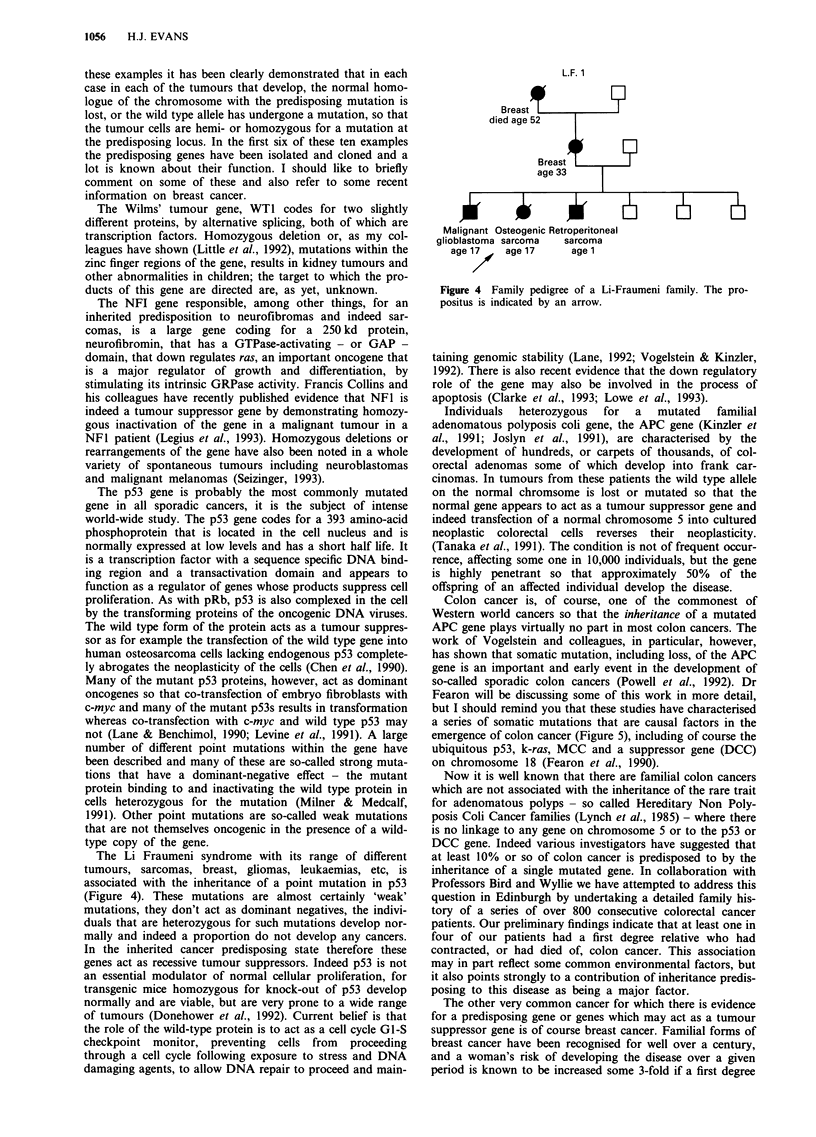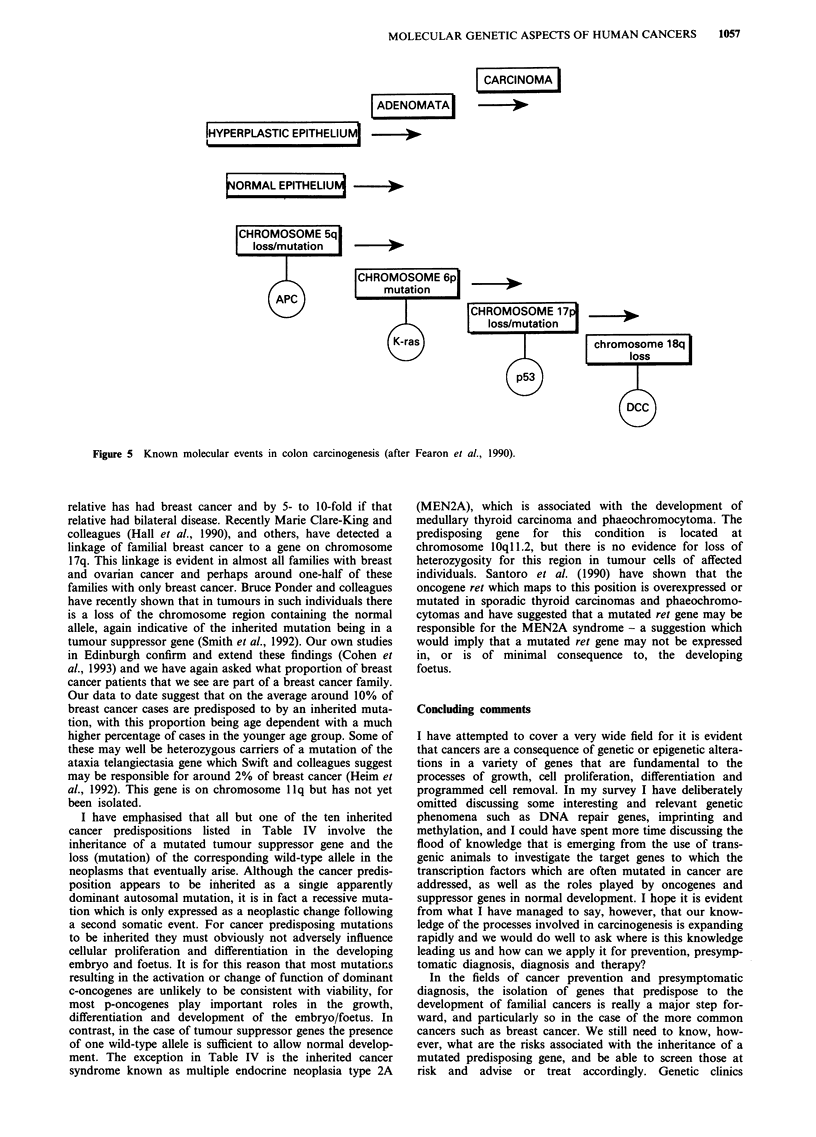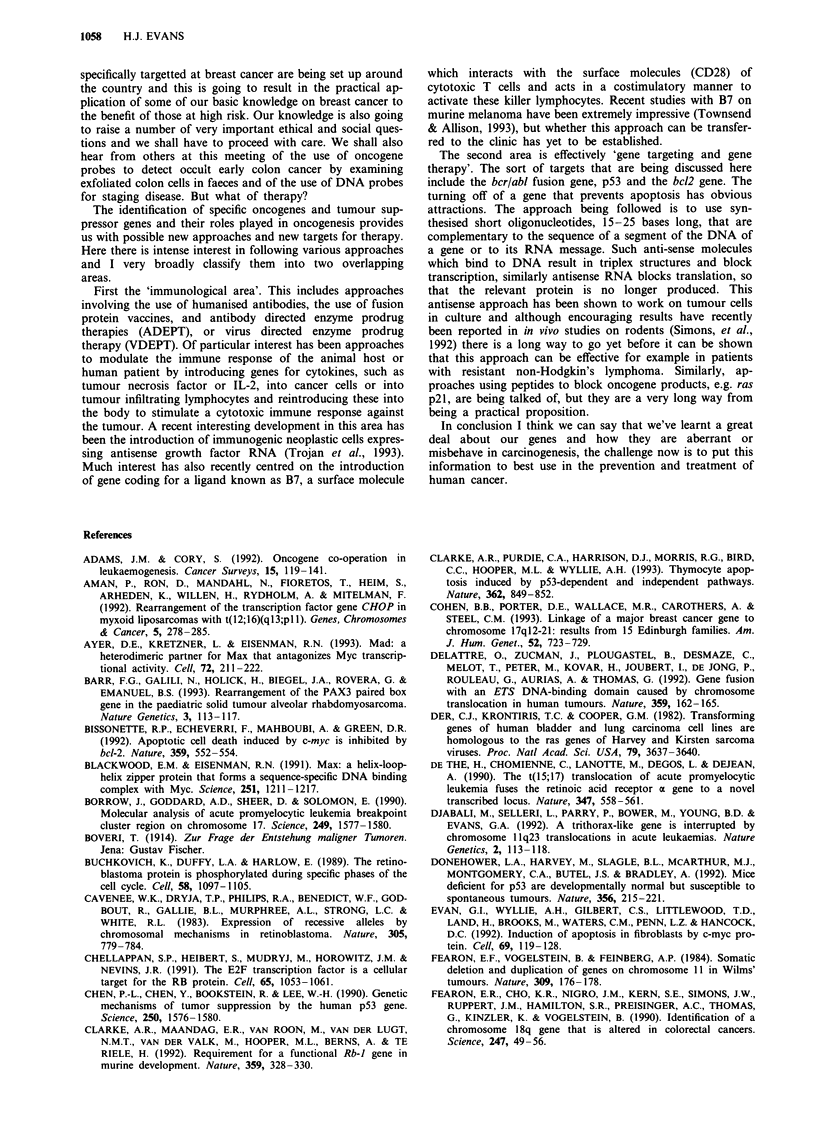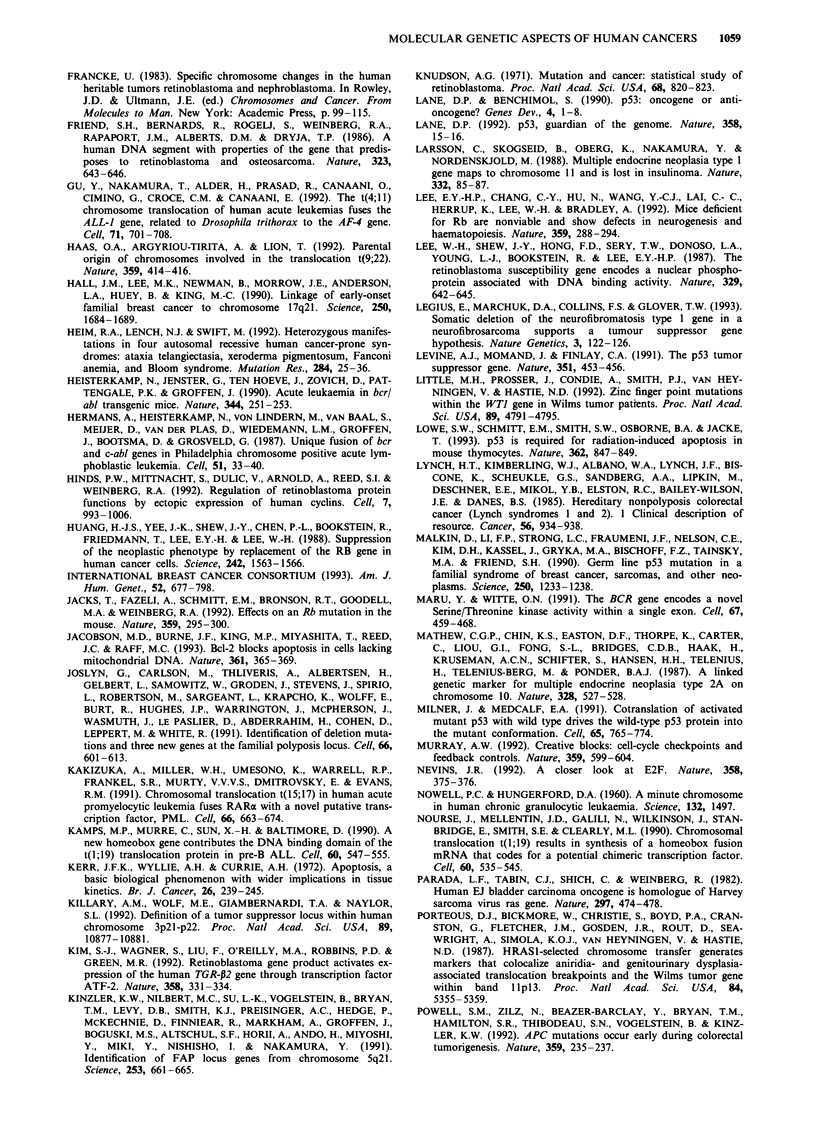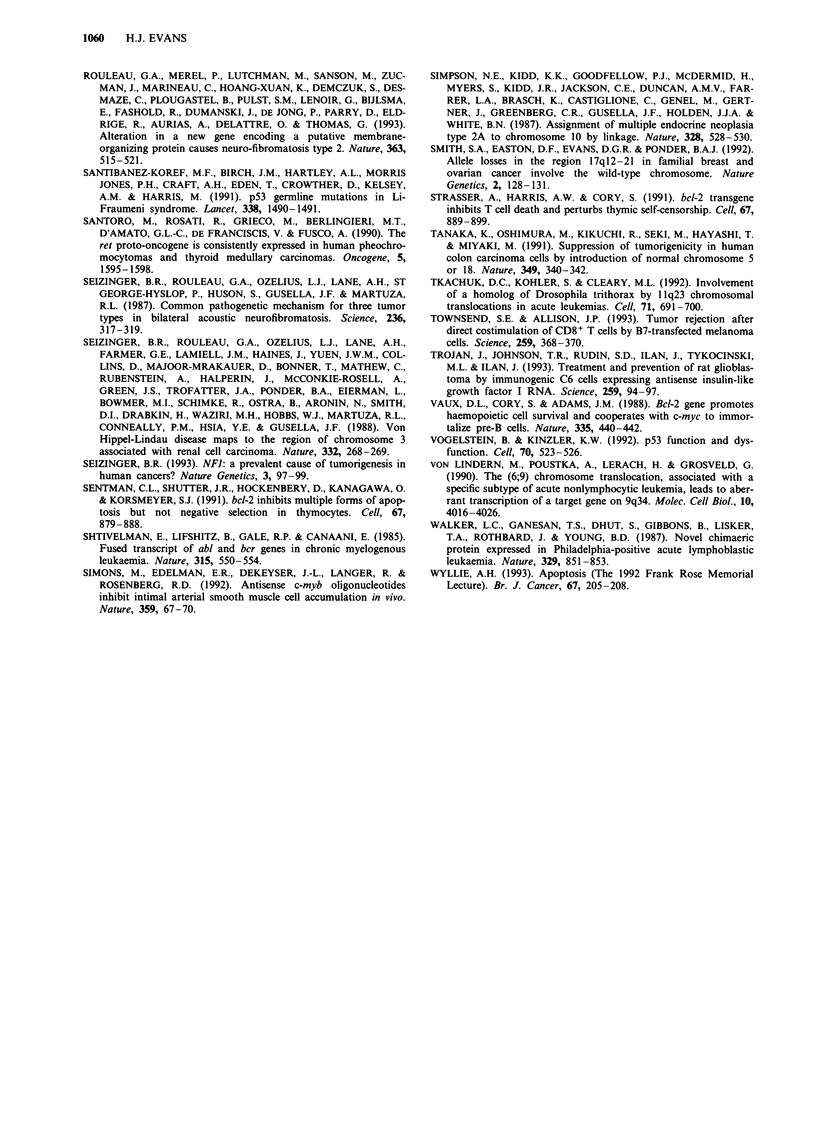# Molecular genetic aspects of human cancers: the 1993 Frank Rose Lecture.

**DOI:** 10.1038/bjc.1993.482

**Published:** 1993-12

**Authors:** H. J. Evans

**Affiliations:** MRC Human Genetics Unit, Western General Hospital, Edinburgh, UK.

## Abstract

**Images:**


*Br. J. Cancer* (1993), **68**, 1051–1060 © Macmillan Press Ltd., 1993

**REVIEW**

### Molecular genetic aspects of human cancers: The 1993 Frank Rose Lecture

H.J. Evans

*MRC Human Genetics Unit, Western General Hospital, Edinburgh, UK.*

The molecular genetics of human cancers clearly covers a very wide field. In this review therefore, I will aim to provide a broad perspective, whilst at the same time attempt to highlight those areas where remarkable advances have been made and those where they are perhaps to be expected.

I should begin by saying that there is no reason to doubt that cancer, or cancers, are very much environmentally triggered diseases, as for example we see in the association between cigarette smoking and lung cancer, but there is also no reason to doubt that cancer is also very much a genetic disease. The notion that cancers are genetic diseases goes back to the turn of the century and in particular to the work of Theodore Boveri (1914) who, on the basis of his studies on abnormalities of growth and development in sea urchins, concluded that abnormal growth resulting in cancers in animals were a direct consequence of the occurrence of chromosome abnormalities. The jump from sea urchins to mammals is in reality a small one, for, as exemplified by more recent molecular genetical studies on such diverse organisms as yeasts, round worms, fruit flies and humans, the genes that we now know are controlling elements in cellular proliferation, in differentiation, in programmed cell death (apoptosis) and organogenesis, have elements in common in all of these species. It has also been known from early studies by Paton Rous and others for example, and later by Bradford Hill and Richard Doll, that carcinogenesis is a multi-stage process which involves the occurrence of at least two and possibly as many as four or even more independent events, a realisation that has been amply confirmed by more recent molecular studies.

The common human cancers that are of major concern are cancers that involve epithelial cells such as those lining the gut, the lungs, cells in the breast and the outer surface layers of the body. These tissues comprise, and their functions are dependent on, normal proliferating cells, as indeed is the case in the bone marrow, and the emergence of a cancer is of course a consequence of a failure to control cell proliferation. The tissues I am referring to have a very high cellular turnover rate, for example our dermatologist colleagues tell us that we completely change our skins once every 6 weeks or so and there is evidence that we shed and replace some 10,000 million epithelial cells from our colons daily and that we destroy and replace some 300,000 million blood and lymphatic cells every 24 h.

The cancers that arise in these tissues are a consequence of a permanent change, or changes, resulting in an abnormally continued, or indeed enhanced, proliferative state. We can look upon these changes as being positive dominant changes driving the cell into an infinite succession of proliferative cycles; or as negative changes which result in a failure on the part of the cell to undergo a normal differentiation or a normal programmed cell death (Figure 1). Each of these processes of proliferation, differentiation and programmed cell death is now clearly known to be governed by a host of families of specific genes, but the first evidence of a genomic mutation that was associated with a specific human malignancy dates back to the work of Peter Nowell and David Hungerford in Philadelphia in 1960 and their discovery of the so-called Philadelphia or Ph[1] chromosome.

Nowell and Hungerford (1960) discovered the consistent presence of an altered small chromosome, the Ph[1] chromosome, in the malignant cells of patients with chronic myeloid leukaemia (CML), this mutation being absent in the normal cells of such patients (Figure 2). We now know that this small abnormal chromosome No 22 is the result of a reciprocal translocation of a terminal part of chromosome 9 onto the end of chromosome 22 and the transfer of a slightly smaller terminal region of chromosome 22 onto chromosome 9 (Figure 3).

The 9/22 translocation in CML involves a breakage at the 5′ end of a specific gene, the *abl* oncogene, on chromosome 9 and the transference of that gene to a breakage site at a gene called the *bcr* (breakage cluster region) gene on chromosome 22. The normal product of the *abl* gene is a 145 kd protein which has tyrosine kinase activity and is expressed in proliferating cells where it is involved in metabolic pathways leading to proliferation. The *abl* gene was originally identified as the direct counterpart of the oncogenic transforming gene of the acutely transforming Ableson leukaemia retrovirus, a virus that produces leukaemia in mice. During its evolution the virus picked up this gene from a mammal and incorporated it into its own genome. The viral form of the gene, *v-abl*, is strongly oncogenic in mice, but the normal form of the *abl* gene in humans, a proto- or p-oncogene is a gene that is essential for normal cellular activity. The translocation in CML results in a mutated cellular oncogene (c-oncogene) which is an extended gene that is transcribed into a 210 kd protein. It has recently been shown that sequences within the first exon of the *bcr* gene are essential for the oncogene activity of the fusion protein and that this gene has serine/threonine kinase activity and can phosphorylate several different protein substrates (Maru & Witte, 1991). It appears

**Figure 1** The pathway from stem cells through proliferation to differentiation and apoptosis.

Received and accepted 27 July 1993.

**Figure 2** Lymphocyte metaphase and karyotype from a patient with CML showing the t9/22 resulting in the Ph[1] chromosome.

therefore that both the *abl* gene and the *bcr* gene may be involved in a number of intra-cellular signalling pathways and the upshot of the translocation that brings these two genes into juxtaposition is that there is a greatly enhanced tyrosine kinase activity relative to that observed in normal cells, which then serves to drive these cells containing the translocation into continued proliferation.

An abnormal *abl* gene is found in all CML cells, but a Ph[1] chromosome is also found in the malignant cells in around 10% of patients with ALL. Here the translocation is at a slightly different site resulting in a somewhat different fusion protein with a molecular weight of 190 kd, again having abnormal tyrosine kinase activity and being strongly autophosphorylated.

The fact that the *abl* fusion protein acts in a dominant oncogenic fashion has been underlined by the work of Heisterkamp and colleagues (1990) who made mice transgenic for a *bcr/abl* P190 DNA, i.e. the DNA that codes for the abnormal gene in ALL. It transpired that these mice, in which the expression of the *bcr/abl* construct was under the control of the *bcr* gene promotor, all died during early embryogenesis. However, when the construct was placed under the control of the metallothionein-1 promotor, a promotor for a housekeeping gene, the gene was expressed in all tissues and eight out of ten surviving transgenic mice died or were moribund with acute leukaemia between 10 and 28 days after birth.

One other recent finding of genetic interest in relation to the *bcr/abl* translocation is that in 11 cases where the parental origin of the two involved chromosomes could be unambiguously ascertained, in all instances the chromosome 9 (*abl* gene) involved the paternally derived chromosome and the chromosome 22 (*bcr* gene) the maternally derived homologue (Haas *et al.*, 1992). This entirely non-random involvement is also evident in relation to certain inherited tumour predispositions and is a phenomenon of considerable current interest that is associated with inherited genomic imprinting.

Since the discovery of the *bcr/abl* gene, a series of examples of translocations resulting in the formation of chimaeric genes which are activated to an oncogenic status have been described and representatives of these are listed in Table I. It turns out that all of these result in fusion proteins which are transcription factors. In other words, they have two important functional regions. One of these is a specific DNA binding site, a zinc finger or helix-turn-helix region, so that the protein binds to a specific DNA target site. The other is a protein binding site, a lucine zipper, which allows dimerisation with another specific protein that functions as a modulator of transcription. Some of these transcription factors may act in one or both of two ways, as for example is the case for the Kruppel gene in *Drosophila* which codes for a zinc finger protein. On the one hand the transcription factor may serve to specifically activate a gene, or genes, required for differentiation and on the other may act as a specific repressor of genes required to be expressed in order to maintain a proliferative state.

**Figure 3** The translocation in CML and ALL resulting in the bcr-abl fusion gene.

An interesting example of a fusion gene is that seen in the malignant cells of around 30% of children with pre-B ALL and which involves the t1/19 translocation. The translocation brings together the PBX-1 gene with its homeobox DNA binding domain on chromosome 1, and which is probably an early development gene, and the E2A transcription factor on chromosome 19 that binds specifically to the enhancer sequence within the immunoglobulin *k* complex (Kamps *et al.*, 1990; Nourse *et al.*, 1990). The PBX-1 gene is not normally expressed in lymphocytes, but its DNA binding domain replaces that on E2A so that the powerful positive signal for lg production is directed at the gene targeted by the PBX-1 protein so that this gene is expressed at the wrong time and in the wrong place.

Before leaving our discussion of chromosome translocations that result in essential oncogene mutations in lymphoid cells, I should like also to point out that there is a whole range of other specific translocations in lymphoid cells which may not, as such, result in mutation in an important regulatory gene, but rather affect its regulation by placing that gene under the control of the promotor of a normally very active gene in that cell. These translocations are a consequence of errors in the DNA processing by the recombinase enzyme during the process of normal reshuffling of the V, D or J regions of the immunoglobulin or T-cell receptor genes (Table II). The classic example here is Burkitt's lymphoma which is associated with one of three different translocations involving chromosome 8 and one of the three immunoglobulin clusters. The consequence of any one of these translocations is to place the *myc* oncogene on chromosome 8 juxtaposed to, and under the control of, the immunoglobulin gene enhancers. Burkitt's lymphoma is a B-cell neoplasm and a major function of B-cells is to produce immunoglobulins so the lg genes are very active. *Myc* on the other hand is an early growth response gene. It codes for a nuclear located protein with a DNA binding helix-loop-helix motif and is a transcription factor. Its lucine zipper region binds to another protein called *max* and the *myc/max* heterodimer binds to a palindromic hexamer sequence, CACGTG which is found in the 5′ region of many genes (Blackwood & Eisenman, 1991; Ayer *et al.*, 1993). The important fact is that the *myc* gene turns on a series of genes that are involved in the proliferation process, so that *myc* is turned on in proliferating cells and turned off in quiescent cells.

A second translocation of particular interest is that found in around 85% of follicular B-cell lymphomas and involves

**Table I** Some examples of translocations resulting in fusion genes in human malignancies

| *Malignancy (translocation)* | *Genes involved* | *Gene structure/function* | *Authors* |
|---|---|---|---|
| CML/ALL t(9q/22q) | 9-*abl* 22-*bcr* | Chimaeric, enhanced TK activity | Shtivelman *et al.* (1985) Hermans *et al.* (1987) Walker *et al.* (1987) |
| ALL t(1q/19p) | 1-PBX-1 19-E2A | Chimaeric TF | Kamps *et al.* (1990) Nourse *et al.* (1990) |
| APML t(15q/17q) | 15-PML 17-RAR | Chimaeric TF | Borrow *et al.* (1990) de The *et al.* (1990) Kakizuka *et al.* (1991) |
| AML t(6p/9q) | 6-CAN | Chimaeric TF | von Lindern *et al.* (1990) |
| ALL/AML t(14q/11q) t(*n*/11q) | 11-H-TRX-1 | Chimaeric TF (Drosoph trithorax) | Djabali *et al.* (1992) Gu *et al.* (1992) Tkachuk *et al.* (1992) |
| Ewings sarcoma t(11q/22q) | 11-HUM FLI-1 22-EWS | Chimaeric TF | Delattre *et al.* (1992) |
| Alveolar rhabdomyosarcoma t(2q/13q) | 2-PAX3 13-? | Chimaeric TF (Waadenburg syndr) | Barr *et al.* (1993) |
| Mixoid liposarcomas t(12q/16p) | 123-CHOP 16-? | Chimaeric TF | Aman *et al.* (1992) |

**Table II** Chromosome translocations and oncogene activations following aberrant DNA processing in lymphoid cells

| *Neoplasm* | *Translocation* | *Affected genes* | *p-oncogene function* |
|---|---|---|---|
| B-ALL | 14q32:8q24 | IgH:cMYC | Transcription factor: cell cycle |
| Burkitt's lymphoma | 2p12:8q24 | Igk:cMYC | ditto |
| | 22q11:8q24 | Igλ:cMYC | ditto |
| B-CLL | 14q32:18q21 | IgH:BCL-2 | anti-apoptosis: prolongation of survival |
| B-lymphoma | 14q32:19q31 | IgH:BCL-3 | protein-protein interaction (= cdc20): cell cycle |
| pre B-ALL | 14q32:5q31 | IgH:IL-3 | Interleukin 3-lymphokine |
| T-ALL | 14q11:1p32 | TCR-α/δ:TAL1 | |
| | 14q11:8q24 | TCR-α/δ:c-MYC | DNA/protein binding |
| | 14q11:10q24 | TCR-α/δ:HOX11 | transcription factors |
| | 14q11:11p13 | TCR-α/δ:Ttg2 | |
| | 14q11:11p15 | TCR-α/δ:Ttg1 | |
| | 7q35:1p32 | TCR-β:TAL1 | |
| | 7q35:9q34 | TCR-β:TAL2 | |
| | 7q35:11p13 | TCR-β:Ttg2 | |
| | 7q35:19p13 | TCR-β:LYL1 | |

the IgH cluster on chromosome 14q32.3, but in this case translocated to a site on chromosome 18q21. The gene at this site, the *bcl2* gene, codes for a small GTP-binding protein which is located on the surfaces of the nuclear membrane and the mitochondria (Jacobson *et al.*, 1993). The association of this *bcl2* gene with the J segment of IgH results in an overproduction of the *bcl2* protein. Normal B cells, of course, ultimately undergo apoptosis i.e. a form of programmed, or physiological, cell death (Kerr *et al.*, 1972) which is characterised by endonuclease mediated DNA fragmentation and chromosome condensation and depends upon RNA and protein synthesis in the dying cell (Wyllie, 1993). It is therefore an active cell-destruct process that is a consequence of the cell's genetic constitution and its reaction to the environment. It transpires, however, that the *bcl2* protein actively prevents cell death and in the presence of an activated *myc* oncogene results in the continuous proliferation on the part of the B cells (Vaux *et al.*, 1988). The activated *bcl2* gene therefore acts as an oncogene and indeed the insertion of a *bcl2*/Ig construct into a transgenic mouse results in extending B cell survival in the living animal and in the formation of follicular lymphomas (Adams & Corey, 1992). This gene therefore exerts its effects through promoting clonal expansion by inhibiting cell death in a population of cells that is normally destined to die (Sentman *et al.*, 1991; Strasser *et al.*, 1991).

The effect of the active *bcl* gene in preventing programmed cell death is evident not only in lymphoid cells, but also in other cell types such as fibroblasts. Studies by Evan *et al.* (1992) and Bissonnette *et al.* (1992) have shown that if c-*myc* is turned on in cultured fibroblasts in the presence of growth factors and in the presence or absence of *bcl2*, the cells undergo continual proliferation in culture. Similar proliferation is observed if c-*myc* and *bcl2* are turned on but growth factors are absent. However, in the absence of growth factors and *bcl2* the cells undergo an apoptosis.

Now I have deliberately introduced the findings on activation of genes to what is essentially a dominant oncogenic state as a result of chromosome translocation using the lymphoid cells as a model, since these cells are readily amenable to chromosome study and enable us to pinpoint the sites of mutation by cytogenetic means. However, I must emphasise that the first isolation and characterisation of cellular oncogenes was from solid tumours such as, for example, the Harvey-*ras* oncogene from human bladder carcinoma cells (Parada *et al.*, 1982; Der *et al.*, 1982). Since these original studies there have been a large number of demonstrations utilising *in vitro* cell transformation assays to isolate a variety of proto-oncogenes that have mutated to a transforming c-oncogenic form in various cell types. These studies have identified various families of genes which are involved as growth factors, growth factor receptors, second messengers and transcription factors, all of which are involved in the processes leading to cellular proliferation (Table III). As a result of a great deal of work now on yeast cells and on mammalian cells in culture, we have also now identified a whole range of genes involved in the mitotic process (Murray, 1992). These genes are under the control of other genes and it is a mutation, or epigenetic change, in these controlling oncogenic genes, following exposure to carcinogenic agents, that are the important initiating factors in carcinogenesis. These studies also serve to underline the fact that in order to produce a malignant transformation it is necessary to have mutations or alterations in expression of more than one gene. As indicated earlier the carcinogenic process requires more than one independent genetic, or epigenetic, event.

So far I have spent my time talking essentially about genes whose altered expression acts in a dominant fashion in driving cells through a continued proliferative state. There are, however, other genes, so-called tumour suppressor or anti-oncogenes, which have the reverse effect, namely to prevent cells proceeding through a proliferative cycle and it has increasingly become evident that these genes play a very important role in carcinogenesis.

The first direct evidence that there were such genes as tumour suppressor genes came from early studies by Henry Harris and colleagues such as Harold Klinger and Eric Stanbridge who showed that the tumorigenicity, in mice, of a neoplastic human cell with an activated oncogene could be suppressed if the neoplastic cell was fused to a normal cell. The fact that such hybrid cells do not give rise to tumours shows that the action of the active oncogene is suppressed by one or more introduced normal genes. Now in such hybrid cells there is usually a loss of donor chromosomes as cells are maintained in culture, so that the cell, or rather its descen-

**Table III** Biological functions of some cellular proto-oncogenes

| *Function* | *Proto-ongogenes* |
|---|---|
| Growth factor | *sis* (PDGF), *int*-2, *hst*-1 |
| Growth factor receptor with TK activity | *erb*B (EGF receptor), *fms* (CSF receptor), *met* (HGF receptor), *neu*, *ros*, *trk*, *ret* |
| Tyrosine kinase | *src*, *abl*, *lck*, *yes* |
| Signal transduction regulation | Ha-*ras*, K-*ras*, N-*ras*, *gsp*, *gip* |
| Serine/threonine kinase | *mos*, *raf* |
| Transcription factors | *myc*, *myb*, *fox*, c-*jun*, *rel* |

dants, eventually become neoplastic once more. Studies on such cells containing the H-*ras*1 activated oncogene show that neoplasticity is reacquired when the hybrid cell loses its normal chromosome 11s, so that the effective suppressor gene must be located on chromosome 11 for this particular tumour. In more recent studies various workers have introduced a specific gene into a range of neoplastic cells and shown that some of these genes can completely abrogate the tumorigenic status of the recipient cell. These studies have been undertaken on a variety of different tumour types, and I shall return briefly to some of these shortly.

Going back in historical time I should say that our first clue to the importance of tumour suppressor genes in human carcinogenesis stemmed from our studies on the rarer familial childhood cancers and, in particular, and at least initially, from studies on retinoblastoma (Knudson, 1971). I would like to briefly summarise these studies as they provide a stepping stone to discuss some of the more recent findings that have been made in our understanding of tumour suppressor genes and inherited cancer predispositions in the context of the more common human cancers.

Retinoblastoma affects about one in every 18,000 children and in around 10% of cases it would appear to be inherited. When it is inherited usually both eyes are affected and there are often multifocal tumours. In the other 90% of cases the tumours are sporadic and here usually only one eye is affected and usually only with one tumour. If we look at the chromosomes of normal blood or skin cells of individuals with inherited retinoblastoma then we often find that we see a small constitutional deletion of part of chromosome 13 at the q14 locus. In contrast, normal somatic cells of people with sporadic retinoblastoma have normal chromosomes, but if we look at the tumour in these people we can often find the same small deletion of chromosome 13q14 that we see in the blood cells of those with the inherited form of the disease (Francke, 1983).

These general features of retinoblastoma are entirely in line with an hypothesis put forward by Alfred Knudson about 20 years ago that in an inherited RB a mutational event is present in, and is transmitted by, the germ line, so that the mutation is present in all the derived somatic cells of the individual. A second mutation then occurs at the same locus in the homologous chromosome in the retinoblast and this results in a recessive change in the RB locus and the development of a tumour. This second mutation may well be, and often is, a complete loss of the chromosome bearing the wild type allele. In sporadic RB both mutations will be somatic acquired mutations. Knudson's hypothesis has been amply confirmed (Cavenee *et al.*, 1983) and the RB gene has now been cloned and characterised (Friend *et al.*, 1986; Lee *et al.*, 1987). It is a very interesting gene whose product is cyclically phosphorylated through the action of cyclins (Hinds *et al.*, 1992) and can be shown to act as a negative controller of cellular proliferation. It is in an unphosphorylated form in $G_0/G_1$ and becomes phosphorylated in the S-phase of the cell cycle (Buchkovich *et al.*, 1989). In its unphosphorylated form it binds to one of the proteins involved in cycling the cells into mitosis, the E2F transcription factor (Chellappan *et al.*, 1991). This transcription factor activates genes involved in DNA synthesis such as DHFR, TK and the DNA $\alpha$ polymerase and the sequestering of E2F by pRb prevents entry into S (Nevins, 1992). The absence of Rb, or presence in an abnormal form, thus prevents it from exerting its down-regulating effect on the cell cycle. Rb has also been implicated as interacting along the pathway involving c-*myc* and also TGF-$\beta$1 and $\beta$2 in proliferating cells and with *myo*-D in the differentiation of muscle cells (Kim *et al.*, 1992; Gu *et al.*, 1992). More recently three groups (Clarke *et al.*, 1992; Jacks *et al.*, 1992; Lee *et al.*, 1992) have succeeded in producing transgenic mice heterozygous for a mutation at the RB-1 locus and these mice develop pituitary tumours rather than retinoblastomas. Embryos homozygous for the mutation are inviable, but the transfer of a normal human RB-1 gene into these embryos corrects the developmental defects.

To underline the importance of the RB mutation I should point out that it is not merely confined to tumours of the retina or to osteosarcomas, but it may also occur as an important mutation in a variety of other human neoplasms. That the gene is indeed a tumour suppressor gene is evident by the fact that it is usually absent or abnormal and inactive in tumour cells. More direct evidence has been provided in the transgenesis studies and by Huang and colleagues (1988) who have directly demonstrated that the neoplastic phenotype of retinoblastoma cells can be suppressed by the introduction of a normal RB gene into these cells.

The retinoblastoma story is a paradigm indicating how the inheritance of a defective tumour suppressor gene is responsible for a familial cancer and it has become evident that this pattern is common to a range of other inherited cancer predispositions. Table IV lists a number of familial cancers which appear to be predisposed to by the inheritance of a single apparently dominant autosomal gene. In all but one of

**Table IV** Some autosomal dominant inherited cancer predispositions in man

| *Syndrome (gene)* | *Principal tumour site/tissue* | *Chromosome and locus* | *Locus deletion in tumour* | *Authors* |
|---|---|---|---|---|
| Retinoblastoma (RB1) | Retina, mesenchymal, osteosarcoma | 13q14 | + | Cavenee *et al.* (1983)<br>Friend *et al.* (1986) |
| Wilms' tumour (WT1) | Kidney | 11p13 | + | Fearon *et al.* (1984)<br>Porteous *et al.* (1987) |
| Familial adenomatous polyposis (APC) | Colorectal adenocarcinoma | 5q21-q22 | + | Kinzler *et al.* (1991) |
| von Recklinghausen neurofibromatosis (NF1) | Neurofibromas, gliomas | 17q11.2 | + | Legius *et al.* (1993) |
| Bilateral acoustic neurofibromatosis (NF2) | Acoustic neuroma, gliomas | 22q11.1-q13.1 | + | Seizinger *et al.* (1987)<br>Rouleau *et al.* (1993) |
| Li Fraumeni (p53) | Osteosarcoma, breast, leuk | 17p13.1 | + | Malkin *et al.* (1990)<br>Santibanez-Koref *et al.* (1991) |
| Familial breast and ovarian ca (BRCA21) | Breast ovary | 17q21-23 | + | Hall *et al.* (1990)<br>Int Breast Ca Consort (1993) |
| Von Hippel-Lindau (VHL) | Renal cell ca, CNS, pancreas | 3p14-p21 (3p21?) | + | Seizinger *et al.* (1988)<br>Killary *et al.* (1992) |
| Multiple endocrine neoplasia type I (MEN1) | Pancreas, pituitary | 11q(cent) | + | Larsson *et al.* (1988) |
| Multiple endocrine neoplasia type 2A (MEN2A) | Thyroid carcinoma | 10p11.2-q11.2 | – | Simpson *et al.* (1987)<br>Mathew *et al.* (1987) |

these examples it has been clearly demonstrated that in each case in each of the tumours that develop, the normal homologue of the chromosome with the predisposing mutation is lost, or the wild type allele has undergone a mutation, so that the tumour cells are hemi- or homozygous for a mutation at the predisposing locus. In the first six of these ten examples the predisposing genes have been isolated and cloned and a lot is known about their function. I should like to briefly comment on some of these and also refer to some recent information on breast cancer.

The Wilms' tumour gene, WT1 codes for two slightly different proteins, by alternative splicing, both of which are transcription factors. Homozygous deletion or, as my colleagues have shown (Little *et al.*, 1992), mutations within the zinc finger regions of the gene, results in kidney tumours and other abnormalities in children; the target to which the products of this gene are directed are, as yet, unknown.

The NFI gene responsible, among other things, for an inherited predisposition to neurofibromas and indeed sarcomas, is a large gene coding for a 250 kd protein, neurofibromin, that has a GTPase-activating – or GAP – domain, that down regulates *ras*, an important oncogene that is a major regulator of growth and differentiation, by stimulating its intrinsic GRPase activity. Francis Collins and his colleagues have recently published evidence that NF1 is indeed a tumour suppressor gene by demonstrating homozygous inactivation of the gene in a malignant tumour in a NF1 patient (Legius *et al.*, 1993). Homozygous deletions or rearrangements of the gene have also been noted in a whole variety of spontaneous tumours including neuroblastomas and malignant melanomas (Seizinger, 1993).

The p53 gene is probably the most commonly mutated gene in all sporadic cancers, it is the subject of intense world-wide study. The p53 gene codes for a 393 amino-acid phosphoprotein that is located in the cell nucleus and is normally expressed at low levels and has a short half life. It is a transcription factor with a sequence specific DNA binding region and a transactivation domain and appears to function as a regulator of genes whose products suppress cell proliferation. As with pRb, p53 is also complexed in the cell by the transforming proteins of the oncogenic DNA viruses. The wild type form of the protein acts as a tumour suppressor as for example the transfection of the wild type gene into human osteosarcoma cells lacking endogenous p53 completely abrogates the neoplasticity of the cells (Chen *et al.*, 1990). Many of the mutant p53 proteins, however, act as dominant oncogenes so that co-transfection of embryo fibroblasts with c-*myc* and many of the mutant p53s results in transformation whereas co-transfection with c-*myc* and wild type p53 may not (Lane & Benchimol, 1990; Levine *et al.*, 1991). A large number of different point mutations within the gene have been described and many of these are so-called strong mutations that have a dominant-negative effect – the mutant protein binding to and inactivating the wild type protein in cells heterozygous for the mutation (Milner & Medcalf, 1991). Other point mutations are so-called weak mutations that are not themselves oncogenic in the presence of a wild-type copy of the gene.

The Li Fraumeni syndrome with its range of different tumours, sarcomas, breast, gliomas, leukaemias, etc, is associated with the inheritance of a point mutation in p53 (Figure 4). These mutations are almost certainly 'weak' mutations, they don't act as dominant negatives, the individuals that are heterozygous for such mutations develop normally and indeed a proportion do not develop any cancers. In the inherited cancer predisposing state therefore these genes act as recessive tumour suppressors. Indeed p53 is not an essential modulator of normal cellular proliferation, for transgenic mice homozygous for knock-out of p53 develop normally and are viable, but are very prone to a wide range of tumours (Donehower *et al.*, 1992). Current belief is that the role of the wild-type protein is to act as a cell cycle G1-S checkpoint monitor, preventing cells from proceeding through a cell cycle following exposure to stress and DNA damaging agents, to allow DNA repair to proceed and maintaining genomic stability (Lane, 1992; Vogelstein & Kinzler, 1992). There is also recent evidence that the down regulatory role of the gene may also be involved in the process of apoptosis (Clarke *et al.*, 1993; Lowe *et al.*, 1993).

**Figure 4** Family pedigree of a Li-Fraumeni family. The propositus is indicated by an arrow.

Individuals heterozygous for a mutated familial adenomatous polyposis coli gene, the APC gene (Kinzler *et al.*, 1991; Joslyn *et al.*, 1991), are characterised by the development of hundreds, or carpets of thousands, of colorectal adenomas some of which develop into frank carcinomas. In tumours from these patients the wild type allele on the normal chromsome is lost or mutated so that the normal gene appears to act as a tumour suppressor gene and indeed transfection of a normal chromosome 5 into cultured neoplastic colorectal cells reverses their neoplasticity. (Tanaka *et al.*, 1991). The condition is not of frequent occurrence, affecting some one in 10,000 individuals, but the gene is highly penetrant so that approximately 50% of the offspring of an affected individual develop the disease.

Colon cancer is, of course, one of the commonest of Western world cancers so that the *inheritance* of a mutated APC gene plays virtually no part in most colon cancers. The work of Vogelstein and colleagues, in particular, however, has shown that somatic mutation, including loss, of the APC gene is an important and early event in the development of so-called sporadic colon cancers (Powell *et al.*, 1992). Dr Fearon will be discussing some of this work in more detail, but I should remind you that these studies have characterised a series of somatic mutations that are causal factors in the emergence of colon cancer (Figure 5), including of course the ubiquitous p53, k-*ras*, MCC and a suppressor gene (DCC) on chromosome 18 (Fearon *et al.*, 1990).

Now it is well known that there are familial colon cancers which are not associated with the inheritance of the rare trait for adenomatous polyps – so called Hereditary Non Polyposis Coli Cancer families (Lynch *et al.*, 1985) – where there is no linkage to any gene on chromosome 5 or to the p53 or DCC gene. Indeed various investigators have suggested that at least 10% or so of colon cancer is predisposed to by the inheritance of a single mutated gene. In collaboration with Professors Bird and Wyllie we have attempted to address this question in Edinburgh by undertaking a detailed family history of a series of over 800 consecutive colorectal cancer patients. Our preliminary findings indicate that at least one in four of our patients had a first degree relative who had contracted, or had died of, colon cancer. This association may in part reflect some common environmental factors, but it also points strongly to a contribution of inheritance predisposing to this disease as being a major factor.

The other very common cancer for which there is evidence for a predisposing gene or genes which may act as a tumour suppressor gene is of course breast cancer. Familial forms of breast cancer have been recognised for well over a century, and a woman's risk of developing the disease over a given period is known to be increased some 3-fold if a first degree

**Figure 5** Known molecular events in colon carcinogenesis (after Fearon *et al.*, 1990).

relative has had breast cancer and by 5- to 10-fold if that relative had bilateral disease. Recently Marie Clare-King and colleagues (Hall *et al.*, 1990), and others, have detected a linkage of familial breast cancer to a gene on chromosome 17q. This linkage is evident in almost all families with breast and ovarian cancer and perhaps around one-half of these families with only breast cancer. Bruce Ponder and colleagues have recently shown that in tumours in such individuals there is a loss of the chromosome region containing the normal allele, again indicative of the inherited mutation being in a tumour suppressor gene (Smith *et al.*, 1992). Our own studies in Edinburgh confirm and extend these findings (Cohen *et al.*, 1993) and we have again asked what proportion of breast cancer patients that we see are part of a breast cancer family. Our data to date suggest that on the average around 10% of breast cancer cases are predisposed to by an inherited mutation, with this proportion being age dependent with a much higher percentage of cases in the younger age group. Some of these may well be heterozygous carriers of a mutation of the ataxia telangiectasia gene which Swift and colleagues suggest may be responsible for around 2% of breast cancer (Heim *et al.*, 1992). This gene is on chromosome 11q but has not yet been isolated.

I have emphasised that all but one of the ten inherited cancer predispositions listed in Table IV involve the inheritance of a mutated tumour suppressor gene and the loss (mutation) of the corresponding wild-type allele in the neoplasms that eventually arise. Although the cancer predisposition appears to be inherited as a single apparently dominant autosomal mutation, it is in fact a recessive mutation which is only expressed as a neoplastic change following a second somatic event. For cancer predisposing mutations to be inherited they must obviously not adversely influence cellular proliferation and differentiation in the developing embryo and foetus. It is for this reason that most mutations resulting in the activation or change of function of dominant c-oncogenes are unlikely to be consistent with viability, for most p-oncogenes play important roles in the growth, differentiation and development of the embryo/foetus. In contrast, in the case of tumour suppressor genes the presence of one wild-type allele is sufficient to allow normal development. The exception in Table IV is the inherited cancer syndrome known as multiple endocrine neoplasia type 2A (MEN2A), which is associated with the development of medullary thyroid carcinoma and phaeochromocytoma. The predisposing gene for this condition is located at chromosome 10q11.2, but there is no evidence for loss of heterozygosity for this region in tumour cells of affected individuals. Santoro *et al.* (1990) have shown that the oncogene *ret* which maps to this position is overexpressed or mutated in sporadic thyroid carcinomas and phaeochromocytomas and have suggested that a mutated *ret* gene may be responsible for the MEN2A syndrome – a suggestion which would imply that a mutated *ret* gene may not be expressed in, or is of minimal consequence to, the developing foetus.

### Concluding comments

I have attempted to cover a very wide field for it is evident that cancers are a consequence of genetic or epigenetic alterations in a variety of genes that are fundamental to the processes of growth, cell proliferation, differentiation and programmed cell removal. In my survey I have deliberately omitted discussing some interesting and relevant genetic phenomena such as DNA repair genes, imprinting and methylation, and I could have spent more time discussing the flood of knowledge that is emerging from the use of transgenic animals to investigate the target genes to which the transcription factors which are often mutated in cancer are addressed, as well as the roles played by oncogenes and suppressor genes in normal development. I hope it is evident from what I have managed to say, however, that our knowledge of the processes involved in carcinogenesis is expanding rapidly and we would do well to ask where is this knowledge leading us and how can we apply it for prevention, presymptomatic diagnosis, diagnosis and therapy?

In the fields of cancer prevention and presymptomatic diagnosis, the isolation of genes that predispose to the development of familial cancers is really a major step forward, and particularly so in the case of the more common cancers such as breast cancer. We still need to know, however, what are the risks associated with the inheritance of a mutated predisposing gene, and be able to screen those at risk and advise or treat accordingly. Genetic clinics specifically targetted at breast cancer are being set up around the country and this is going to result in the practical application of some of our basic knowledge on breast cancer to the benefit of those at high risk. Our knowledge is also going to raise a number of very important ethical and social questions and we shall have to proceed with care. We shall also hear from others at this meeting of the use of oncogene probes to detect occult early colon cancer by examining exfoliated colon cells in faeces and of the use of DNA probes for staging disease. But what of therapy?

The identification of specific oncogenes and tumour suppressor genes and their roles played in oncogenesis provides us with possible new approaches and new targets for therapy. Here there is intense interest in following various approaches and I very broadly classify them into two overlapping areas.

First the 'immunological area'. This includes approaches involving the use of humanised antibodies, the use of fusion protein vaccines, and antibody directed enzyme prodrug therapies (ADEPT), or virus directed enzyme prodrug therapy (VDEPT). Of particular interest has been approaches to modulate the immune response of the animal host or human patient by introducing genes for cytokines, such as tumour necrosis factor or IL-2, into cancer cells or into tumour infiltrating lymphocytes and reintroducing these into the body to stimulate a cytotoxic immune response against the tumour. A recent interesting development in this area has been the introduction of immunogenic neoplastic cells expressing antisense growth factor RNA (Trojan *et al*., 1993). Much interest has also recently centred on the introduction of gene coding for a ligand known as B7, a surface molecule which interacts with the surface molecules (CD28) of cytotoxic T cells and acts in a costimulatory manner to activate these killer lymphocytes. Recent studies with B7 on murine melanoma have been extremely impressive (Townsend & Allison, 1993), but whether this approach can be transferred to the clinic has yet to be established.

The second area is effectively 'gene targeting and gene therapy'. The sort of targets that are being discussed here include the *bcr/abl* fusion gene, p53 and the *bcl2* gene. The turning off of a gene that prevents apoptosis has obvious attractions. The approach being followed is to use synthesised short oligonucleotides, 15–25 bases long, that are complementary to the sequence of a segment of the DNA of a gene or to its RNA message. Such anti-sense molecules which bind to DNA result in triplex structures and block transcription, similarly antisense RNA blocks translation, so that the relevant protein is no longer produced. This antisense approach has been shown to work on tumour cells in culture and although encouraging results have recently been reported in *in vivo* studies on rodents (Simons, *et al*., 1992) there is a long way to go yet before it can be shown that this approach can be effective for example in patients with resistant non-Hodgkin's lymphoma. Similarly, approaches using peptides to block oncogene products, e.g. *ras* p21, are being talked of, but they are a very long way from being a practical proposition.

In conclusion I think we can say that we've learnt a great deal about our genes and how they are aberrant or misbehave in carcinogenesis, the challenge now is to put this information to best use in the prevention and treatment of human cancer.

### References

ADAMS, J.M. & CORY, S. (1992). Oncogene co-operation in leukaemogenesis. *Cancer Surveys*, **15**, 119–141.

AMAN, P., RON, D., MANDAHL, N., FIORETOS, T., HEIM, S., ARHEDEN, K., WILLEN, H., RYDHOLM, A. & MITELMAN, F. (1992). Rearrangement of the transcription factor gene *CHOP* in myxoid liposarcomas with t(12;16)(q13;p11). *Genes, Chromosomes & Cancer*, **5**, 278–285.

AYER, D.E., KRETZNER, L. & EISENMAN, R.N. (1993). Mad: a heterodimeric partner for Max that antagonizes Myc transcriptional activity. *Cell*, **72**, 211–222.

BARR, F.G., GALILI, N., HOLICK, H., BIEGEL, J.A., ROVERA, G. & EMANUEL, B.S. (1993). Rearrangement of the PAX3 paired box gene in the paediatric solid tumour alveolar rhabdomyosarcoma. *Nature Genetics*, **3**, 113–117.

BISSONETTE, R.P., ECHEVERRI, F., MAHBOUBI, A. & GREEN, D.R. (1992). Apoptotic cell death induced by c-*myc* is inhibited by *bcl*-2. *Nature*, **359**, 552–554.

BLACKWOOD, E.M. & EISENMAN, R.N. (1991). Max: a helix-loop-helix zipper protein that forms a sequence-specific DNA binding complex with Myc. *Science*, **251**, 1211–1217.

BORROW, J., GODDARD, A.D., SHEER, D. & SOLOMON, E. (1990). Molecular analysis of acute promyelocytic leukemia breakpoint cluster region on chromosome 17. *Science*, **249**, 1577–1580.

BOVERI, T. (1914). *Zur Frage der Entstehung maligner Tumoren.* Jena: Gustav Fischer.

BUCHKOVICH, K., DUFFY, L.A. & HARLOW, E. (1989). The retinoblastoma protein is phosphorylated during specific phases of the cell cycle. *Cell*, **58**, 1097–1105.

CAVENEE, W.K., DRYJA, T.P., PHILIPS, R.A., BENEDICT, W.F., GODBOUT, R., GALLIE, B.L., MURPHREE, A.L., STRONG, L.C. & WHITE, R.L. (1983). Expression of recessive alleles by chromosomal mechanisms in retinoblastoma. *Nature*, **305**, 779–784.

CHELLAPPAN, S.P., HEIBERT, S., MUDRYJ, M., HOROWITZ, J.M. & NEVINS, J.R. (1991). The E2F transcription factor is a cellular target for the RB protein. *Cell*, **65**, 1053–1061.

CHEN, P.-L., CHEN, Y., BOOKSTEIN, R. & LEE, W.-H. (1990). Genetic mechanisms of tumor suppression by the human p53 gene. *Science*, **250**, 1576–1580.

CLARKE, A.R., MAANDAG, E.R., VAN ROON, M., VAN DER LUGT, N.M.T., VAN DER VALK, M., HOOPER, M.L., BERNS, A. & TE RIELE, H. (1992). Requirement for a functional *Rb-1* gene in murine development. *Nature*, **359**, 328–330.

CLARKE, A.R., PURDIE, C.A., HARRISON, D.J., MORRIS, R.G., BIRD, C.C., HOOPER, M.L. & WYLLIE, A.H. (1993). Thymocyte apoptosis induced by p53-dependent and independent pathways. *Nature*, **362**, 849–852.

COHEN, B.B., PORTER, D.E., WALLACE, M.R., CAROTHERS, A. & STEEL, C.M. (1993). Linkage of a major breast cancer gene to chromosome 17q12-21: results from 15 Edinburgh families. *Am. J. Hum. Genet.*, **52**, 723–729.

DELATTRE, O., ZUCMAN, J., PLOUGASTEL, B., DESMAZE, C., MELOT, T., PETER, M., KOVAR, H., JOUBERT, I., DE JONG, P., ROULEAU, G., AURIAS, A. & THOMAS, G. (1992). Gene fusion with an *ETS* DNA-binding domain caused by chromosome translocation in human tumours. *Nature*, **359**, 162–165.

DER, C.J., KRONTIRIS, T.C. & COOPER, G.M. (1982). Transforming genes of human bladder and lung carcinoma cell lines are homologous to the ras genes of Harvey and Kirsten sarcoma viruses. *Proc. Natl Acad. Sci. USA*, **79**, 3637–3640.

DE THE, H., CHOMIENNE, C., LANOTTE, M., DEGOS, L. & DEJEAN, A. (1990). The t(15;17) translocation of acute promyelocytic leukemia fuses the retinoic acid receptor α gene to a novel transcribed locus. *Nature*, **347**, 558–561.

DJABALI, M., SELLERI, L., PARRY, P., BOWER, M., YOUNG, B.D. & EVANS, G.A. (1992). A trithorax-like gene is interrupted by chromosome 11q23 translocations in acute leukaemias. *Nature Genetics*, **2**, 113–118.

DONEHOWER, L.A., HARVEY, M., SLAGLE, B.L., MCARTHUR, M.J., MONTGOMERY, C.A., BUTEL, J.S. & BRADLEY, A. (1992). Mice deficient for p53 are developmentally normal but susceptible to spontaneous tumours. *Nature*, **356**, 215–221.

EVAN, G.I., WYLLIE, A.H., GILBERT, C.S., LITTLEWOOD, T.D., LAND, H., BROOKS, M., WATERS, C.M., PENN, L.Z. & HANCOCK, D.C. (1992). Induction of apoptosis in fibroblasts by c-myc protein. *Cell*, **69**, 119–128.

FEARON, E.F., VOGELSTEIN, B. & FEINBERG, A.P. (1984). Somatic deletion and duplication of genes on chromosome 11 in Wilms' tumours. *Nature*, **309**, 176–178.

FEARON, E.R., CHO, K.R., NIGRO, J.M., KERN, S.E., SIMONS, J.W., RUPPERT, J.M., HAMILTON, S.R., PREISINGER, A.C., THOMAS, G., KINZLER, K. & VOGELSTEIN, B. (1990). Identification of a chromosome 18q gene that is altered in colorectal cancers. *Science*, **247**, 49–56.

FRANCKE, U. (1983). Specific chromosome changes in the human heritable tumors retinoblastoma and nephroblastoma. In Rowley, J.D. & Ultmann, J.E. (ed.) *Chromosomes and Cancer. From Molecules to Man*. New York: Academic Press, p. 99–115.

FRIEND, S.H., BERNARDS, R., ROGELJ, S., WEINBERG, R.A., RAPAPORT, J.M., ALBERTS, D.M. & DRYJA, T.P. (1986). A human DNA segment with properties of the gene that predisposes to retinoblastoma and osteosarcoma. *Nature*, **323**, 643–646.

GU, Y., NAKAMURA, T., ALDER, H., PRASAD, R., CANAANI, O., CIMINO, G., CROCE, C.M. & CANAANI, E. (1992). The t(4;11) chromosome translocation of human acute leukemias fuses the *ALL-1* gene, related to *Drosophila trithorax* to the *AF-4* gene. *Cell*, **71**, 701–708.

HAAS, O.A., ARGYRIOU-TIRITA, A. & LION, T. (1992). Parental origin of chromosomes involved in the translocation t(9;22). *Nature*, **359**, 414–416.

HALL, J.M., LEE, M.K., NEWMAN, B., MORROW, J.E., ANDERSON, L.A., HUEY, B. & KING, M.-C. (1990). Linkage of early-onset familial breast cancer to chromosome 17q21. *Science*, **250**, 1684–1689.

HEIM, R.A., LENCH, N.J. & SWIFT, M. (1992). Heterozygous manifestations in four autosomal recessive human cancer-prone syndromes: ataxia telangiectasia, xeroderma pigmentosum, Fanconi anemia, and Bloom syndrome. *Mutation Res.*, **284**, 25–36.

HEISTERKAMP, N., JENSTER, G., TEN HOEVE, J., ZOVICH, D., PATTENGALE, P.K. & GROFFEN, J. (1990). Acute leukaemia in *bcr/abl* transgenic mice. *Nature*, **344**, 251–253.

HERMANS, A., HEISTERKAMP, N., VON LINDERN, M., VAN BAAL, S., MEIJER, D., VAN DER PLAS, D., WIEDEMANN, L.M., GROFFEN, J., BOOTSMA, D. & GROSVELD, G. (1987). Unique fusion of *bcr* and c-*abl* genes in Philadelphia chromosome positive acute lymphoblastic leukemia. *Cell*, **51**, 33–40.

HINDS, P.W., MITTNACHT, S., DULIC, V., ARNOLD, A., REED, S.I. & WEINBERG, R.A. (1992). Regulation of retinoblastoma protein functions by ectopic expression of human cyclins. *Cell*, **7**, 993–1006.

HUANG, H.-J.S., YEE, J.-K., SHEW, J.-Y., CHEN, P.-L., BOOKSTEIN, R., FRIEDMANN, T., LEE, E.Y.-H. & LEE, W.-H. (1988). Suppression of the neoplastic phenotype by replacement of the RB gene in human cancer cells. *Science*, **242**, 1563–1566.

INTERNATIONAL BREAST CANCER CONSORTIUM (1993). *Am. J. Hum. Genet.*, **52**, 677–798.

JACKS, T., FAZELI, A., SCHMITT, E.M., BRONSON, R.T., GOODELL, M.A. & WEINBERG, R.A. (1992). Effects on an *Rb* mutation in the mouse. *Nature*, **359**, 295–300.

JACOBSON, M.D., BURNE, J.F., KING, M.P., MIYASHITA, T., REED, J.C. & RAFF, M.C. (1993). Bcl-2 blocks apoptosis in cells lacking mitochondrial DNA. *Nature*, **361**, 365–369.

JOSLYN, G., CARLSON, M., THLIVERIS, A., ALBERTSEN, H., GELBERT, L., SAMOWITZ, W., GRODEN, J., STEVENS, J., SPIRIO, L., ROBERTSON, M., SARGEANT, L., KRAPCHO, K., WOLFF, E., BURT, R., HUGHES, J.P., WARRINGTON, J., MCPHERSON, J., WASMUTH, J., LE PASLIER, D., ABDERRAHIM, H., COHEN, D., LEPPERT, M. & WHITE, R. (1991). Identification of deletion mutations and three new genes at the familial polyposis locus. *Cell*, **66**, 601–613.

KAKIZUKA, A., MILLER, W.H., UMESONO, K., WARRELL, R.P., FRANKEL, S.R., MURTY, V.V.V.S., DMITROVSKY, E. & EVANS, R.M. (1991). Chromosomal translocation t(15;17) in human acute promyelocytic leukemia fuses RARα with a novel putative transcription factor, PML. *Cell*, **66**, 663–674.

KAMPS, M.P., MURRE, C., SUN, X.-H. & BALTIMORE, D. (1990). A new homeobox gene contributes the DNA binding domain of the t(1;19) translocation protein in pre-B ALL. *Cell*, **60**, 547–555.

KERR, J.F.K., WYLLIE, A.H. & CURRIE, A.H. (1972). Apoptosis, a basic biological phenomenon with wider implications in tissue kinetics. *Br. J. Cancer*, **26**, 239–245.

KILLARY, A.M., WOLF, M.E., GIAMBERNARDI, T.A. & NAYLOR, S.L. (1992). Definition of a tumor suppressor locus within human chromosome 3p21-p22. *Proc. Natl Acad. Sci. USA*, **89**, 10877–10881.

KIM, S.-J., WAGNER, S., LIU, F., O'REILLY, M.A., ROBBINS, P.D. & GREEN, M.R. (1992). Retinoblastoma gene product activates expression of the human *TGR-β2* gene through transcription factor ATF-2. *Nature*, **358**, 331–334.

KINZLER, K.W., NILBERT, M.C., SU, L.-K., VOGELSTEIN, B., BRYAN, T.M., LEVY, D.B., SMITH, K.J., PREISINGER, A.C., HEDGE, P., MCKECHNIE, D., FINNIEAR, R., MARKHAM, A., GROFFEN, J., BOGUSKI, M.S., ALTSCHUL, S.F., HORII, A., ANDO, H., MIYOSHI, Y., MIKI, Y., NISHISHO, I. & NAKAMURA, Y. (1991). Identification of FAP locus genes from chromosome 5q21. *Science*, **253**, 661–665.

KNUDSON, A.G. (1971). Mutation and cancer: statistical study of retinoblastoma. *Proc. Natl Acad. Sci. USA*, **68**, 820–823.

LANE, D.P. & BENCHIMOL, S. (1990). p53: oncogene or anti-oncogene? *Genes Dev.*, **4**, 1–8.

LANE, D.P. (1992). p53, guardian of the genome. *Nature*, **358**, 15–16.

LARSSON, C., SKOGSEID, B., OBERG, K., NAKAMURA, Y. & NORDENSKJOLD, M. (1988). Multiple endocrine neoplasia type 1 gene maps to chromosome 11 and is lost in insulinoma. *Nature*, **332**, 85–87.

LEE, E.Y.-H.P., CHANG, C.-Y., HU, N., WANG, Y.-C.J., LAI, C.- C., HERRUP, K., LEE, W.-H. & BRADLEY, A. (1992). Mice deficient for Rb are nonviable and show defects in neurogenesis and haematopoiesis. *Nature*, **359**, 288–294.

LEE, W.-H., SHEW, J.-Y., HONG, F.D., SERY, T.W., DONOSO, L.A., YOUNG, L.-J., BOOKSTEIN, R. & LEE, E.Y.-H.P. (1987). The retinoblastoma susceptibility gene encodes a nuclear phosphoprotein associated with DNA binding activity. *Nature*, **329**, 642–645.

LEGIUS, E., MARCHUK, D.A., COLLINS, F.S. & GLOVER, T.W. (1993). Somatic deletion of the neurofibromatosis type 1 gene in a neurofibrosarcoma supports a tumour suppressor gene hypothesis. *Nature Genetics*, **3**, 122–126.

LEVINE, A.J., MOMAND, J. & FINLAY, C.A. (1991). The p53 tumor suppressor gene. *Nature*, **351**, 453–456.

LITTLE, M.H., PROSSER, J., CONDIE, A., SMITH, P.J., VAN HEYNINGEN, V. & HASTIE, N.D. (1992). Zinc finger point mutations within the *WT1* gene in Wilms tumor patients. *Proc. Natl Acad. Sci. USA*, **89**, 4791–4795.

LOWE, S.W., SCHMITT, E.M., SMITH, S.W., OSBORNE, B.A. & JACKE, T. (1993). p53 is required for radiation-induced apoptosis in mouse thymocytes. *Nature*, **362**, 847–849.

LYNCH, H.T., KIMBERLING, W.J., ALBANO, W.A., LYNCH, J.F., BISCONE, K., SCHEUKLE, G.S., SANDBERG, A.A., LIPKIN, M., DESCHNER, E.E., MIKOL, Y.B., ELSTON, R.C., BAILEY-WILSON, J.E. & DANES, B.S. (1985). Hereditary nonpolyposis colorectal cancer (Lynch syndromes 1 and 2). 1 Clinical description of resource. *Cancer*, **56**, 934–938.

MALKIN, D., LI, F.P., STRONG, L.C., FRAUMENI, J.F., NELSON, C.E., KIM, D.H., KASSEL, J., GRYKA, M.A., BISCHOFF, F.Z., TAINSKY, M.A. & FRIEND, S.H. (1990). Germ line p53 mutation in a familial syndrome of breast cancer, sarcomas, and other neoplasms. *Science*, **250**, 1233–1238.

MARU, Y. & WITTE, O.N. (1991). The *BCR* gene encodes a novel Serine/Threonine kinase activity within a single exon. *Cell*, **67**, 459–468.

MATHEW, C.G.P., CHIN, K.S., EASTON, D.F., THORPE, K., CARTER, C., LIOU, G.I., FONG, S.-L., BRIDGES, C.D.B., HAAK, H., KRUSEMAN, A.C.N., SCHIFTER, S., HANSEN, H.H., TELENIUS, H., TELENIUS-BERG, M. & PONDER, B.A.J. (1987). A linked genetic marker for multiple endocrine neoplasia type 2A on chromosome 10. *Nature*, **328**, 527–528.

MILNER, J. & MEDCALF, E.A. (1991). Cotranslation of activated mutant p53 with wild type drives the wild-type p53 protein into the mutant conformation. *Cell*, **65**, 765–774.

MURRAY, A.W. (1992). Creative blocks: cell-cycle checkpoints and feedback controls. *Nature*, **359**, 599–604.

NEVINS, J.R. (1992). A closer look at E2F. *Nature*, **358**, 375–376.

NOWELL, P.C. & HUNGERFORD, D.A. (1960). A minute chromosome in human chronic granulocytic leukaemia. *Science*, **132**, 1497.

NOURSE, J., MELLENTIN, J.D., GALILI, N., WILKINSON, J., STANBRIDGE, E., SMITH, S.E. & CLEARLY, M.L. (1990). Chromosomal translocation t(1;19) results in synthesis of a homeobox fusion mRNA that codes for a potential chimeric transcription factor. *Cell*, **60**, 535–545.

PARADA, L.F., TABIN, C.J., SHICH, C. & WEINBERG, R. (1982). Human EJ bladder carcinoma oncogene is homologue of Harvey sarcoma virus ras gene. *Nature*, **297**, 474–478.

PORTEOUS, D.J., BICKMORE, W., CHRISTIE, S., BOYD, P.A., CRANSTON, G., FLETCHER, J.M., GOSDEN, J.R., ROUT, D., SEAWRIGHT, A., SIMOLA, K.O.J., VAN HEYNINGEN, V. & HASTIE, N.D. (1987). HRAS1-selected chromosome transfer generates markers that colocalize aniridia- and genitourinary dysplasia-associated translocation breakpoints and the Wilms tumor gene within band 11p13. *Proc. Natl Acad. Sci. USA*, **84**, 5355–5359.

POWELL, S.M., ZILZ, N., BEAZER-BARCLAY, Y., BRYAN, T.M., HAMILTON, S.R., THIBODEAU, S.N., VOGELSTEIN, B. & KINZLER, K.W. (1992). *APC* mutations occur early during colorectal tumorigenesis. *Nature*, **359**, 235–237.

ROULEAU, G.A., MEREL, P., LUTCHMAN, M., SANSON, M., ZUCMAN, J., MARINEAU, C., HOANG-XUAN, K., DEMCZUK, S., DESMAZE, C., PLOUGASTEL, B., PULST, S.M., LENOIR, G., BIJLSMA, E., FASHOLD, R., DUMANSKI, J., DE JONG, P., PARRY, D., ELDRIGE, R., AURIAS, A., DELATTRE, O. & THOMAS, G. (1993). Alteration in a new gene encoding a putative membrane-organizing protein causes neuro-fibromatosis type 2. *Nature*, **363**, 515–521.

SANTIBANEZ-KOREF, M.F., BIRCH, J.M., HARTLEY, A.L., MORRIS JONES, P.H., CRAFT, A.H., EDEN, T., CROWTHER, D., KELSEY, A.M. & HARRIS, M. (1991). p53 germline mutations in Li-Fraumeni syndrome. *Lancet*, **338**, 1490–1491.

SANTORO, M., ROSATI, R., GRIECO, M., BERLINGIERI, M.T., D'AMATO, G.L.-C., DE FRANCISCIS, V. & FUSCO, A. (1990). The *ret* proto-oncogene is consistently expressed in human pheochromocytomas and thyroid medullary carcinomas. *Oncogene*, **5**, 1595–1598.

SEIZINGER, B.R., ROULEAU, G.A., OZELIUS, L.J., LANE, A.H., ST GEORGE-HYSLOP, P., HUSON, S., GUSELLA, J.F. & MARTUZA, R.L. (1987). Common pathogenetic mechanism for three tumor types in bilateral acoustic neurofibromatosis. *Science*, **236**, 317–319.

SEIZINGER, B.R., ROULEAU, G.A., OZELIUS, L.J., LANE, A.H., FARMER, G.E., LAMIELL, J.M., HAINES, J., YUEN, J.W.M., COLLINS, D., MAJOOR-MRAKAUER, D., BONNER, T., MATHEW, C., RUBENSTEIN, A., HALPERIN, J., MCCONKIE-ROSELL, A., GREEN, J.S., TROFATTER, J.A., PONDER, B.A., EIERMAN, L., BOWMER, M.I., SCHIMKE, R., OSTRA, B., ARONIN, N., SMITH, D.I., DRABKIN, H., WAZIRI, M.H., HOBBS, W.J., MARTUZA, R.L., CONNEALLY, P.M., HSIA, Y.E. & GUSELLA, J.F. (1988). Von Hippel-Lindau disease maps to the region of chromosome 3 associated with renal cell carcinoma. *Nature*, **332**, 268–269.

SEIZINGER, B.R. (1993). *NF1*: a prevalent cause of tumorigenesis in human cancers? *Nature Genetics*, **3**, 97–99.

SENTMAN, C.L., SHUTTER, J.R., HOCKENBERY, D., KANAGAWA, O. & KORSMEYER, S.J. (1991). *bcl*-2 inhibits multiple forms of apoptosis but not negative selection in thymocytes. *Cell*, **67**, 879–888.

SHTIVELMAN, E., LIFSHITZ, B., GALE, R.P. & CANAANI, E. (1985). Fused transcript of *abl* and *bcr* genes in chronic myelogenous leukaemia. *Nature*, **315**, 550–554.

SIMONS, M., EDELMAN, E.R., DEKEYSER, J.-L., LANGER, R. & ROSENBERG, R.D. (1992). Antisense c-*myb* oligonucleotides inhibit intimal arterial smooth muscle cell accumulation *in vivo*. *Nature*, **359**, 67–70.

SIMPSON, N.E., KIDD, K.K., GOODFELLOW, P.J., MCDERMID, H., MYERS, S., KIDD, J.R., JACKSON, C.E., DUNCAN, A.M.V., FARRER, L.A., BRASCH, K., CASTIGLIONE, C., GENEL, M., GERTNER, J., GREENBERG, C.R., GUSELLA, J.F., HOLDEN, J.J.A. & WHITE, B.N. (1987). Assignment of multiple endocrine neoplasia type 2A to chromosome 10 by linkage. *Nature*, **328**, 528–530.

SMITH, S.A., EASTON, D.F., EVANS, D.G.R. & PONDER, B.A.J. (1992). Allele losses in the region 17q12–21 in familial breast and ovarian cancer involve the wild-type chromosome. *Nature Genetics*, **2**, 128–131.

STRASSER, A., HARRIS, A.W. & CORY, S. (1991). *bcl*-2 transgene inhibits T cell death and perturbs thymic self-censorship. *Cell*, **67**, 889–899.

TANAKA, K., OSHIMURA, M., KIKUCHI, R., SEKI, M., HAYASHI, T. & MIYAKI, M. (1991). Suppression of tumorigenicity in human colon carcinoma cells by introduction of normal chromosome 5 or 18. *Nature*, **349**, 340–342.

TKACHUK, D.C., KOHLER, S. & CLEARY, M.L. (1992). Involvement of a homolog of Drosophila trithorax by 11q23 chromosomal translocations in acute leukemias. *Cell*, **71**, 691–700.

TOWNSEND, S.E. & ALLISON, J.P. (1993). Tumor rejection after direct costimulation of $CD8^+$ T cells by B7-transfected melanoma cells. *Science*, **259**, 368–370.

TROJAN, J., JOHNSON, T.R., RUDIN, S.D., ILAN, J., TYKOCINSKI, M.L. & ILAN, J. (1993). Treatment and prevention of rat glioblastoma by immunogenic C6 cells expressing antisense insulin-like growth factor I RNA. *Science*, **259**, 94–97.

VAUX, D.L., CORY, S. & ADAMS, J.M. (1988). *Bcl*-2 gene promotes haemopoietic cell survival and cooperates with c-*myc* to immortalize pre-B cells. *Nature*, **335**, 440–442.

VOGELSTEIN, B. & KINZLER, K.W. (1992). p53 function and dysfunction. *Cell*, **70**, 523–526.

VON LINDERN, M., POUSTKA, A., LERACH, H. & GROSVELD, G. (1990). The (6;9) chromosome translocation, associated with a specific subtype of acute nonlymphocytic leukemia, leads to aberrant transcription of a target gene on 9q34. *Molec. Cell Biol.*, **10**, 4016–4026.

WALKER, L.C., GANESAN, T.S., DHUT, S., GIBBONS, B., LISKER, T.A., ROTHBARD, J. & YOUNG, B.D. (1987). Novel chimaeric protein expressed in Philadelphia-positive acute lymphoblastic leukaemia. *Nature*, **329**, 851–853.

WYLLIE, A.H. (1993). Apoptosis (The 1992 Frank Rose Memorial Lecture). *Br. J. Cancer*, **67**, 205–208.